# Strategy Use Among Chinese as Second Language Learners in Mainland China From the Mediation Theory Perspective

**DOI:** 10.3389/fpsyg.2021.752084

**Published:** 2021-10-13

**Authors:** Chili Li, Lu Chen, Chunyan Ma, Shuang Zhang, Haiquan Huang

**Affiliations:** ^1^School of Foreign Languages, Hubei University of Technology, Wuhan, China; ^2^International College, Jiangxi University of Engineering, Xinyu, China; ^3^Department of Education, Faculty of Arts and Social Sciences, New Era University College, Kajang, Malaysia; ^4^Department of Chinese Language and Literature, Faculty of Arts and Social Sciences, New Era University College, Kajang, Malaysia; ^5^School of Humanities, Wuhan University of Engineering Science, Wuhan, China

**Keywords:** learners of Chinese as second language, use of learning strategies, mediation theory, learner self, socio-cultural factors

## Abstract

This paper reports a mixed-methods study that explored the strategy use of a cohort of Chinese as second language learners in mainland China from the perspective of mediation theory. Data sources include a questionnaire survey (*N* = 189) and a semi-structured interview (*N* = 12). The findings revealed that the participants orchestrated a repertoire of language learning strategies and frequently used social and meta-cognitive strategies. Analysis of the qualitative data suggested that the participants' strategy use was shaped by the learners' self agentic power (their beliefs and Ideal L2 self), and the socio-cultural environment. Specifically, their strategy use was mediated by a host of socio-cultural factors, including learner beliefs, social agents, cultural artifacts, and learning environment. Considered together, the findings illuminate the socially situated nature of the use of language learning strategy. That is, strategy use of the participants stems from the interplay of learner agency and socio-cultural factors. The findings also imply the necessity of strategy-based instruction and highlight the importance of a Chinese-speaking environment for Chinese learning.

## Introduction

The past two decades have witnessed a surging number of people learning Chinese as a second/foreign language (CSL/CFL) in and outside China (Gong et al., 2018, 2020a). It has been reported that more than 20 million people from more than 180 countries/regions were learning Chinese as an additional language by the end of 2020 (Sun, 2021). Around one quarter million international students were learning Chinese in mainland China in 2019 according to a report released by the Ministry of Education (MOE) of China (MOE, 2019). For all the dramatically increasing volume of CSL learners around the world, the learning process of those learners is found to be under-researched (Luo and Sun, 2018). A number of relevant issues warrant further exploration that includes profiling repertoire of strategy use (Bao and Jiang, 2020), and assessing and improving strategic competence in learning Chinese (Bruen, 2020; Yang et al., 2021).

Existing research has explored the orchestration of using learning strategies among learners in various Chinese-as-foreign-language (CFL) settings (i.e., Bruen, 2020; Chen et al., 2021; Yang et al., 2021). These studies mainly documented the use of learning strategies among CFL learners in their home countries. However, relatively few studies have paid attention to the endorsement of learning strategies when an increasing number of Chinese-as-second-language (CSL) learners are being recently relocated into Chinese-speaking contexts like mainland China (Chu et al., 2015; Luo and Sun, 2018).

Previous studies on learning strategies have been mainly cognitively-oriented (Macaro, 2006; Gao, 2010). The cognitive perspective takes learning strategies as a static construct and fails to unveil the influence of the socio-cultural factors upon learners' strategic efforts (Macaro, 2006). Besides, there is an increasing voice that learning strategies should be regarded as a socially situated construct (Gao, 2006) and a shifting educational context would reshape the situatedness of strategy use (Gong et al., 2021a). Echoing this call, the socio-cultural perspective has been proposed as an alternative in learning strategy research (Donato and Mccormick, 1994). This socio-cultural lens to strategy use could present a holistic picture of how the learners develop their linguistic knowledge as well as how they adapt to the target language context (Cohen and Griffiths, 2015; Gong et al., 2020b). However, although the socio-cultural orientation has been drawing attention in the research of learning strategies of English as second/foreign language learners (ESL/EFL) (Donato and Mccormick, 1994), little effort has been made to unveil the underpinning factors that might have formulated the mechanism of using learning strategies among the CSL/CFL learners.

To address the issues aforementioned, the present study aims to investigate the use of learning strategies among a group of international students in mainland China. More specifically, it proposes to explore the mediational process of the use of Chinese language learning strategies. This study promises to illuminate the nature of strategy use of CSL learners, which in return contributes to the existing literature of learning strategies, in particular, Chinese language learning strategies.

## Literature Review

### Strategy Use in Second/Foreign Language Learning

Learning strategy has been regarded as an essential factor in second language learning and acquisition (Griffiths, 2020). Early effort to learning strategy has been dominated by the cognitive perspective. This approach conceptualizes learning strategies into multiple categories, namely, learning, communication, and social strategies (Rubin, 1975), meta-cognitive, cognitive, and social-affective strategies (O'Malley and Chamot, 1990), and the six-category taxonomy of learning strategies (Oxford, 1990). Among these cognition-oriented theoretical proposals, Oxford's (1990) taxonomy is the far-reaching classic for later learning strategy research.

Following the cognitive approach and Oxford's (1990) taxonomy, researchers have extensively examined the use of learning strategies among English as second/foreign language (ESL/EFL) learners of diversified socio-cultural backgrounds (Rao, 2016; Griffiths, 2020). For example, Chinese learners of English are reported to have moderately used learning strategies (Li, 2014). In addition, students of non-English disciplines in EFL context tend to frequently use memory and cognitive strategies, but least deploy affective and social strategies (Rao, 2016). By contrast, this finding is incompatible with the observation that learners in ESL context use meta-cognitive and social strategies the most (Zhong, 2015). Last but not the least, the use of learning strategies is associated with other learner variables such as beliefs (Zhong, 2015) and motivation (Hajar, 2018). For instance, a belief about the primacy of using language for communication seems to shape the learners' more frequent use of social strategies rather than rote learning strategies (Zhong, 2015). Learners' deployment of social strategies is also reported to be influenced by their ideal L2 self (Hajar, 2018).

In summary, the studies reviewed above have deepened our understanding of the features of learning strategies in various socio-cultural contexts, and the relationship between strategy use and other learner variables. However, these previous studies have been primarily confined to ESL/EFL learners. It remains unknown whether these features identified in early studies can be generalized to learners of languages other than English such as Chinese.

### Strategy Use in Chinese Language Learning

Chinese as foreign language (CFL) learners' strategy use has been drawing attention in recent years (Chu et al., 2015). Existing research mainly follows Oxford's (1990) Strategy Inventory for Language Learning (SILL) and her criteria to measure and evaluate the profiles of strategy use among CFL learners. Social strategies were found to be frequently used by CFL learners in various contexts such as Australia (Jiang and Wu, 2016), Spain (Wang and Cáceres-Lorenzo, 2019) and Brazil (Yang et al., 2021). Brazilian beginning CFL learners displayed a high level of employing meta-cognitive strategies, only secondary to social strategies (Yang et al., 2021). Irish CFL learners frequently exercised cognitive strategies (Bruen, 2020), while Spanish CFL learners did not deploy these strategies that much (Wang and Cáceres-Lorenzo, 2019). In addition, it has been reported that affective strategies were favored by the British CFL learners (Qian et al., 2018), but least frequently deployed by their Australian counterparts (Jiang and Wu, 2016).

Previous research, though scant in number, has started to explore the use of learning strategies among CSL learners. The CSL learners tend to use cognitive and meta-cognitive strategies most frequently in learning Chinese characters (Luo and Sun, 2018; Sheu, 2018). These two categories of learning strategies are also favored by Chinese heritage speakers in their learning of Chinese characters (Bao and Jiang, 2020). To recap, the previous research subjects have mainly included the CFL learners, while inadequate attention has been paid to CSL learners. In light of the increasing number of CSL learners in mainland China, it is essential to explore how these learners adapt themselves to this new context which is linguistically, pedagogically and socially different from the one in their home environments (Ma et al., 2017).

Previous studies have shown that the use of learning strategies seems to be divergent in the CFL and CSL contexts, which sheds light on the contextually situated nature of learning strategy (Li, 2014). However, these studies have mainly followed the cognitive orientation which is increasingly criticized for its overreliance on normative techniques to measure learners' strategy use and for its failure in unveiling the socio-cultural complexity of learning strategies (Lei, 2012). Against this background, it has been advocated to capture the socio-cultural disposition of learning strategies (Gao, 2006).

In a nutshell, the review of the existing research reveals that whilst the early studies have mainly focused on strategy use of CFL learners, they paid inadequate attention to CSL learners. Besides, these previous studies primarily relied on quantitative techniques to examine the features of strategy use among the learners of Chinese, but failed to take into account the interaction of strategy use and social cultural contexts. There is also paucity in profiling strategy use of CSL learners and in understanding the socio-cultural dispositions of their strategy use.

### Strategy Use From a Socio-Cultural Perspective

The socio-cultural perspective conceptualizes human beings' cognitive development as a mediational process in which a host of personal, social, cultural and physical tools are applied by the individual to actualize the meaningfulness of an activity (Vygotsky, 1978; Lantolf, 2000). The mediational perspective is introduced to understand strategy use of second/foreign language learning (Donato and Mccormick, 1994; Gao, 2006).

Following the mediational approach, previous research has mainly focused on strategy use among EFL/ESL learners. For instance, when shifting from mainland China to the UK, Chinese ESL learners come to apply more social strategies due to the new medium language for instruction and communication, academic learning tasks, social agents, learning environment, and learner self (Gao, 2006; Liu, 2013). Learners tend to apply private speech–memorize words (de Guerrero, 2018), control affect state (Jiménez-Jiménez, 2015), and interact with others (Smith, 2007). Assessment practice such as formative methods and portfolio has mediating effect on learners' strategy use (Donato and Mccormick, 1994). Social agents like teachers are also found to be a critical mediator in learners' strategy use (Oxford, 2014).

Taken together, these findings suggest that factors such as agents, assessment methods and learning environment tend to mediate strategy use in learning the target language (Li, 2014; Perea, 2019). These mediational tools encompass learner self associated with beliefs and attitudes toward language learning (Gao, 2006; Lantolf, 2006), social agents such as parents, teachers, and peers (Lantolf, 2000), cultural artifacts pertaining to resources like assessment, textbooks and other learning materials (Lantolf and Poehner, 2008; Gao, 2010), and learning environment at macro and situational levels (Gao, 2010; Lei, 2012). These tools will be used as the guiding analytical framework for the present study. Since previous studies following the mediational lens have mainly centered on ESL/EFL learners, more effort to examine CSL learners' strategy use from this perspective is necessary.

To sum up, the above literature review has shown that the use of learning strategies of CSL learners has not received due attention and that existing research on strategy use fails to take socio-cultural context into account. The inadequacy in focusing on the CSL learners' strategy use and in crystallizing the interaction between strategy use and socio-cultural conditions thus necessitates further exploration. Therefore, to address these gaps this study proposes to explore the strategy use among CSL learners from the perspective of mediation theory.

## RESEARCH DESIGN

### Research Questions

(1) What are the features of learning strategies among the CSL learners?

(2) What mediational factors might have contributed to the use of learning strategies among those CSL learners?

### Research Context and Participants

This study was conducted at a provincial key university in Central China. The university started to recruit international students as early as 1998. Since then, it has a steady annual population of 1,000 international students. It was among the list of the first cohort of institutions of higher education who passed the quality evaluation of international education programmes accredited by the Ministry of Education of China in 2019. It was therefore chosen as the research setting of this study. The university offers the CSL learners a series of Chinese language modules regarding Chinese characters, pronunciation, listening, speaking, reading, culture, and preparatory courses for the Chinese language proficiency test (Hanyu Shuiping Kaoshi: HSK). The preparatory courses are mainly directed at HSK Level 3 for most of the CSL learners at the university. Only a small proportion of them would aspire to sit the HSK Level 4. The CSL learners are required to take these courses for a certain length of time before they are qualified to upgrade onto the degree programs. These courses are delivered by native Chinese-speaking teachers who have obtained postgraduate degrees in the fields of teaching Chinese to speakers of other languages. Formative assessment methods are practiced with such elements as presentations, projects, mid-term, and final exams. Asides from these courses, it provides the learners with some extra-curricular activities including Chinese Calligraphy and costumes, Chinese corner, Taiji, Chinese talent shows and Chinese language proficiency competitions at university, provincial, and national levels.

One hundred eighty-nine CSL learners were involved in the questionnaire survey of this study. These participants included 55 males and 134 females, who were from 26 countries including Morocco, Kazakhstan, Congo, Zimbabwe, Thailand, Russia and others. They ranged in age from 16 to 26, with an average age of 21.37. Among these participants, 90 of them had an experience of learning Chinese <6 months, 80 of them from 6 to 12 months, and 19 of them over 12 months. It is noteworthy that a great majority of these participants had limited experience of learning Chinese before the survey. They may be roughly regarded as beginning learners of CSL.

Twelve students were invited for the semi-structured interviews of this study (Table 1). There were 5 females and 7 males, with four of them from Kazakhstan, two from Russia, and the other six from Algeria, Ghana, Morocco, Laos, Thailand, and Bangladesh, respectively. These interviewees were aged from 18 to 26 years old. Each four of them fell into the three groups of length of time spent learning Chinese, including the <6-month group, the 6- to 12-month group, and the over-12-month group. All the interviewees were pseudonymously named as Ahao, Anan, Benliyi, Chenan, Doha, Hadi, Molly, Qiaozhen, Wangyu, Xiaoye, Xiaoyi, and Yiwan respectively.

**Table 1:** Demographic Information of the Interviewees.

**No**.	**Name**	**Gender**	**Age**	**Nationality**	**Length of time spent learning Chinese**
1	Hadi	Male	26	Bangladesh	12 months
2	Qiaozhen	Female	24	Thailand	24 months
3	Wangyu	Male	21	Laos	18 months
4	Benliyi	Male	23	Algeria	6 months
5	Doha	Female	23	Morocco	6 months
6	Chenan	Male	22	Ghana	5 months
7	Molly	Female	19	Kazakhstan	4 months
8	Yiwan	Male	23	Kazakhstan	10 months
9	Anan	Female	20	Kazakhstan	17 months
10	Xiaoye	Female	18	Kazakhstan	15 months
11	Xiaoyi	Male	21	Russia	12 months
12	Ahao	Male	23	Russia	11 months

### Instruments

#### Questionnaire

A self-designed questionnaire was adapted from Oxford (1990) seminal SILL. Modifications were made in order to situate the questionnaire into the Chinese language learning contexts for this study. For example, the word “*English*” was replaced with “*Chinese*.” For another example, the statement “*I use rhymes to remember new English words*” was changed into “*I use Pinyin to remember newly learned Chinese characters*.” Before the questionnaire was finalized, six CSL learners were invited to participate in a pilot study for the purpose of checking the validity of the instrument. Based on the feedback from the pilot study, further revisions were made accordingly.

The finalized questionnaire consisted of two sections. Section 1 was designed to inquire the demographic information of the participants such as gender, age, nationality, and length of time spent learning Chinese. Section 2 included 53 items, which fell into six categories including Memory Strategies (Item 1–10), Cognitive Strategies (Item 11–26), Compensation Strategies (Item 27–32), Meta-cognitive Strategies (Item 33–41), Affective Strategies (Item 42–47), and Social Strategies (Item 48–53). These items followed a 5-point Likert scale, ranging from *Completely Inapplicable to my Situation* (1) to *Completely Applicable to my Situation* (5). The questionnaire was originally designed with an English-Chinese version, and the Chinese parts were translated into English by a doctoral researcher in applied linguistics. The reliability of the questionnaire was measured with Cronbach Alpha. The Cronbach Alpha for the whole questionnaire is 0.93, with the ones for the six dimensions ranging from 0.715 to 0.853. The results reveal that the questionnaire has good internal consistency and high reliability.

#### Interview Protocol

An interview protocol (Appendix 1) was designed for the semi-structured interview by drawing on sources from previous studies (e.g., Gao, 2010). In so doing, it aimed to obtain in-depth information about the mediational factors that might have shaped the application of learning strategies among the CSL learners. Similar to the designing process of the questionnaire, a pilot study was conducted with 2 of the participants of the present study. Alterations were made according to the feedback from the 2 participants. The finalized protocol embraced two parts. The first part elicited the interviewees' background information such as their name, gender, age, nationality, and length of time of learning Chinese. The second part included aspects such as the strategies they adopted to learn Chinese, the challenges they encountered in their Chinese learning and the ways they used to help themselves out, the impressive Chinese class they ever had, their strategic response to role of examinations, the most influential person in their Chinese learning, and their ways to learn Chinese after class. Each of the aspects was well-attended by asking the interviewees to provide specific examples and reasons.

### Data Collection

Before administering the questionnaire survey, the authors contacted the Chinese language teachers of the CSL learners for their consent of participation. Upon consent obtained, the participants were informed of the objectives of this questionnaire survey. The participants were also told that their responses to the survey would not influence the scores of their final examination and that the collected information would be kept confidentially. Two hundred ten copies of the questionnaire were distributed to the participants, with 206 copies returned. Among the returned ones, 17 copies were taken as invalid because of incompletion or wrongly answered problems. In total, 189 valid copies were included into the final data analysis.

Twelve out of the 189 quantitative respondents were invited for the follow-up semi-structured interviews by following a purposive sampling method (Dörnyei, 2007). The interviews were guided by the interview protocol. All the interviews were conducted in English. Even though Chinese was the target language to the interviewees, in light of their limited Chinese language proficiency as indicated in the length of time they spent on learning Chinese prior to the survey of this study. English was therefore used in the interviews to elicit information on the reasons for their strategy use as much as possible, because it was the common language for communication between the interviewees and the authors of this study. Immediately after each interview, the interviewee's responses were checked by the interviewer to see whether the interviewee had some points to be clarified due to his/her poor English language proficiency. Each interview lasted 30–50 min and was recorded for subsequent coding and data analysis.

### Data Analysis

The data collected from the questionnaire were analyzed by the Statistical Package for the Social Sciences (SPSS 22.0). Descriptive techniques such as means and standard deviation were applied to gain a basic picture of using learning strategies among the CSL learners (Research Question 1).

The interview data were processed with the qualitative data analysis programme ATLAS.ti. In particular, the data were analyzed by means of the qualitative content analysis approach (Dörnyei, 2007) so as to find out the mediational factors that might have influenced the participants' use of learning strategies (Research Question 2). More specifically, the analytical approach followed an iterative process including initial coding, secondary coding, and theorizing the emergent themes. During the initial coding, if there was any account associated with the learners' use of learning strategies, it would be coded. For example, the following account “*My Chinese language teacher often encourages me to communicate with him and other Chinese students so that I come to understand the importance of talking to native Chinese-speakers*” was coded as teacher mediation on learners' use of social strategies. Based on the analysis, a coding scheme was developed by following the above steps (Please refer to **Table 3** in section Factors influencing strategy use among the participants for the details of the coding scheme). In the secondary coding, the data were recoded and re-classified to seek emergent thematic patterns from the initial coding. The newly obtained categories emerging from the secondary coding were repeatedly compared and checked by referring to the mediational framework (Gao, 2010; Lei, 2012). This iterative process finally yielded the reasons for the learners' adoption of learning strategies.

To ensure the coding reliability, one third of the transcribed data were re-classified 3 months after the initial coding. The newly coded results were then compared with those completed earlier. The intra-coder coefficient was 148/167 = 0.886, which suggested that the coding of the data was consistently reliable over time (Miles and Huberman, 1994). Those inconsistent results were further compared and revised until the categorization was finalized.

## Results

### Features of Using Learning Strategies Among the Participants

When analyzing the collected quantitative data to answer Research Question 1 regarding the features of using learning strategies among the participants, this study adopted the judging criteria proposed by Oxford (1990). According to her, the mean of the five-point Likert scale represents a level of using learning strategies, with the value above 3.5 as a high level, the value between 3.0 and 3.5 as an intermediate-upper level, and the value between 2.5 and 3.0 as an intermediate-lower level. However, the value below 2.5 symbolizes a low level. Table 2 reports the level of strategy use in learning Chinese among the participants.

**Table 2:** Level of strategy use in learning Chinese among the participants.

**Dimension**	***N***.	**Min**.	**Max**.	**Mean**	**Standard deviation**
Memory	189	1.0	5.0	3.562	1.0513
Cognitive	189	1.0	5.0	3.467	1.0929
Compensation	189	1.0	5.0	3.437	1.0627
Meta-Cognitive	189	1.0	5.0	3.836	0.9961
Affective	189	1.0	5.0	3.444	1.1217
Social	189	1.0	5.0	3.888	1.0233
Overall	189	1.0	5.0	3.606	1.0580

As depicted in Table 2, the overall mean of the strategy use scale was 3.606, which was above 3.5. This suggested that the participants displayed a high level of deploying learning strategies in general. In addition, the means for social, meta-cognitive and memory strategies were 3.888, 3.836, and 3.562, respectively, all above 3.5. These results revealed that the participants exhibited a high level of using these three dimensional strategies. By contrast, the means for cognitive, affective and compensation strategies were 3.467, 3.444, and 3.437, respectively, all these values falling between 3.0 and 3.5. These results implied that the participants were at an intermediate-upper level in endorsing these three subcategories of learning strategies.

### Factors Influencing Strategy Use Among the Participants

The interview data were analyzed within the mediational framework (Gao, 2010; Lei, 2012), using a qualitative content analysis approach (Dörnyei, 2007). This analysis was made to explore the reasons for the participants' use of learning strategies (Research Question 2). The analysis of the qualitative data yielded four major mediational themes, which encompassed social agents, self mediation, cultural artifacts, and learning environment (Table 3).

**Table 3:** Mediational factors identified from the qualitative data.

**Mediational factors**	**Secondary mediational factors**	**Frequency**
Social agents	Teachers	30
	Peers	45
Learner self	Private speech	61
Cultural artifacts	Medium language	17
	WhatsApp and online resources	39
	Textbooks and other materials	45
	Assessment methods	24
Learning Environment	Macro environment	62
	Classroom environment	31
	Learning community	24

#### Social Agents

Social agents refer to important others including teachers, peers and friends, parents and other family members, who might mediate learners' strategy use in their second/foreign language learning (Gao, 2006; Li, 2014). The interview data revealed that the CSL learners' application of learning strategies was mediated by a number of important others including teachers and peers. A good rapport between teachers and students seems to have mediated the interviewees' deployment of affective strategies. For instance, Interviewee Hadi acknowledged the mediating effect of his teacher on his endorsement of social-affective strategies: “*Well I found most of the Chinese teachers here are quite helpful and very friendly, and they always motivate us to learn in an easier way and give us the tips to learn it*” (Hadi, Extract 1). It was revealed from Hadi's responses that a harmonious teacher-student relationship would promote the learners' recognition of the teacher and his teaching, which in return could stimulate the learners to take initiative in learning the target language. Similar opinion is also concurred by other interviewees like Doha who said that “…* I become more confident to learn Chinese when I feel his (Teacher Alex) approval of my performance*” (Doha, Extract 2). The appreciation of teachers upon the students' performance seems to signal a positive psychological indication to the learner, which in return considerably enhanced their confidence in learning the language.

The interview data also revealed that peers played a critical role in mediating the utilization of social strategies among the interviewees. Remarks like “…* my Chinese friends often help correct my Chinese pronunciations and grammatical mistakes*” (Benliyi, Extract 3) showed that the interviewees would exercise social strategies to seek help from their Chinese peers and friends when encountering difficulties in their Chinese learning. Another peer influence relates to the endorsement of meta-cognitive strategies among the participants. For example, Chenan mentioned: “*Mostly I learned Chinese by myself by writing 5–6 Chinese characters, which were subsequently used in my talk with my Chinese friends*” (Chenan, Extract 4). This shows that the participant bewared how to learn Chinese by exercising his meta-cognition. He initially learned the target language on his/her own and then strengthened the learnt knowledge through interacting with peers.

#### Learner Self

Private speech is often approbated by learners to regulate their language learning (Xu and Fu, 2019). It refers to the behavior of whistling or murmuring to oneself during language learning (Xi, 2020), which contributes to the cognitive development of the learners (de Guerrero and Commander, 2013).

The analysis of the interview data revealed that private speech was applied by the interviewees to mediate their exercise of meta-cognitive strategies. For example, Ahao, a competent learner, said as follows: “*I keep telling myself that I need to fill out all by myself. …I do make plans for what am I going to study Chinese every day*” (Ahao, Extract 5). Such kind of private speech indicated that self reminding is the ground for a learner to take initiative in planning his/her Chinese learning, which reflects the deployment of meta-cognitive strategies. Private speech was also found to have influenced the deployment of affective strategies among the interviewees. As mentioned by Hadi, “… *That is why I always tell myself that for the sake of my mom's devotion to me, I will have to work harder on Chinese*” (Hadi, Extract 6). It was evident that reminding himself of his mother's commitment to him was taken as an encouragement by Hadi in his Chinese learning.

#### Cultural Artifacts

Cultural artifacts are a kind of mediational resources associated with semiotic signs and physical tools (Vygotsky, 1978). Cultural artifacts include linguistic and non-linguistic resources which could promote cognitive development. Mediational resources like mother tongue, the target language, textbooks and online materials have been found to influence learners' strategy use (Lei, 2012; Li, 2014).

The interviews suggested that the medium language of communication was claimed to have a mediating effect upon the learners' employment of compensation strategies. Learners would turn to the commonly accepted medium language in case of a breakdown in their communication. For example, Doha said that “*When people don't understand my Chinese, I would use English (to explain again)*” (Doha, Extract 7). The example indicates that participants like Doha were able to endorse compensation strategies in circumstances in which they encountered breakdowns in an attempt to communicate by utilizing the target language such as Chinese.

Moreover, WhatsApp and other online resources were found to mediate the learners' use of cognitive strategies. For example, Xiaoye mentioned that “*I would go to Youtube to watch Chinese language learning programs when I am learning the tones of Chinese characters*” (Xiaoye, Extract 8). It seems that these learners were familiar with using WhatsApp to facilitate their Chinese language learning. There were various online resources like language learning videos and movies at the university where the interviewees were studying, which can accommodate Chinese learning. This process reflects the role of mediation of WhatsApp in the learners' application of cognitive strategies.

Textbooks and other teaching materials were also reported with a mediational effect upon the deployment of cognitive strategies among the interviewees. As Molly said, “*I love reading textbooks with pictures and pinyin because the pinyin and pictures helps my understanding very easily*” (Molly, Extract 9). Other interviewees like Qiaozhen also similarly expressed that they would resort to textbook exercises to consolidate her Chinese learning, which shows the mediation of textbooks on learners' use of cognitive strategies.

Assessment methods pertain to the ways applied to evaluate learners' performance, which usually encompass summative assessment like final exams and formative assessment such as presentations, essays, projects, and mid-term and final examinations (Li, 2014). Assessment practices like formative assessment and high-stake examinations were found to mediate the learners' exercise of memory strategies. As Molly said, “*I realized I was more attentive to my study before an examination than on ordinary occasions. I would recite and write more characters in order to keep them in my mind*” (Molly, Extract 10). The remark suggested that the participant mainly resorted to memory strategies to prepare for her examinations. In addition, examinations seemed to drive the learners to approbate meta-cognitive strategies. For example, Anan said: “*For exams, I would plan and regularly review once in a while, in case I will repeat my mistakes again”* (Anan, Extract 11). This shows that she has learnt to reflect on all the exercises for better performance in the examinations.

#### Learning Environment

Learning environment includes the contexts for learning at micro level like institutional learning conditions and macro levels such as social environment (Lamb, 2013). Social environment was found to influence the harnessing of social strategies among the participants. For example, Ahao said: “*I learn Chinese because I want to stay here in future. To survive in China requires me to learn Chinese as well as Chinese people. Therefore, I learn it by conversing with the locals around us, which is beneficial for my Chinese learning*” (Ahao, Extract 12). His ideal L2 self seemed to shape his strategic belief that socializing with the locals is effective for learning Chinese.

Besides, social environment might have mediated the participants' employment of meta-cognitive strategies. For example, Yiwan mentioned that he came to know the effectiveness of learning Chinese through talking to local Chinese people. When he tried to greet the local Chinese by saying “*Hello! How are you?”* he found that these people would respond to him with “*Have you had your dinner?”*(Yiwan, Extract 13). Therefore, he came to learn that to utilize Chinese to talk with the locals is more effective than to learn only from the textbooks. His interaction with the local Chinese made him reflect on what he learned from textbook and what he observed in real-life language use, which influenced his choice of strategies to learn Chinese.

Another illustration of the influence of learning environment upon the use of learning strategies of the participants regards class environment. Classroom environment was found to have possibly influenced the learners' approbation of social strategies. As commented by Doha, “*In my class, we have some real good students who can speak very fluent Chinese. So sometimes if I have any problems, I will ask turn to them for help*” (Doha, Extract 14). This is echoed by Anan who voiced that “*I would turn to our Chinese student assistants when I have difficulties in learning Chinese*” (Anan, Extract 15). Availability of more capable learners in class illustrates the mediating effect of classroom environment upon learners' use of social strategy in their Chinese learning.

Lastly, learning associations were found to have probably exerted influence upon the learners' social-affective strategies. Xiaoye expressed that “*I encouraged myself to participate in the Chinese Calligraphy Association, where I could learn from my peers*” (Xiaoye, Extract 16). In short, learning associations provided opportunities for the learners to interact with their Chinese peers so that they could make better achievements in Chinese learning.

## Discussion

The present study yielded two major findings. Firstly, it has identified the commonalities and divergences in strategy use of the participants from learners in other CFL/CSL contexts. The surveyed CSL learners showed a frequent deployment of social, meta-cognitive and memory strategies. Their frequent use of social and meta-cognitive strategies corroborates with the previous findings in both the CFL and CSL contexts (e.g., Luo and Sun, 2018; Chen et al., 2021; Yang et al., 2021). Besides, the present study revealed a moderate level of using affective strategies among the participants, which contradicts the previous findings that the CFL learners frequently use affective strategies in the British context (e.g., Qian et al., 2018). Moreover, the present study distinguishes itself from previous studies in that memory strategies are found to be frequently applied by the CSL learners. This finding has not been previously reported with regard to the strategic profiles of CSL learners, thereby enriching the existing literature. The participants' frequent use of memory strategies might be expounded as follows: for one thing, since most of them were beginning learners, they might rely on direct strategies, memory strategies in particular. Learners of low level of language proficiency are often found to frequently use memory strategies (Yu and Wang, 2009; Griffiths, 2013). For another, the participants surveyed in this study were supposed to pass the HSK test. This might be another reason that drove them to adopt memory strategies in their preparation for the test.

The second major finding is that the participants' strategy use manifested a contextually situated construct, which resulted from the interplay of learner agency and the social and cultural conditions. The present study explored the mediating factors upon the learners' strategy use at personal, situational, institutional, and contextual levels. It has found that learner self is a central mediator in shaping the interviewees' strategy use. Learners like Ahao, Hadi, and Qiaozhen exercised their private speech in learning Chinese. This result accords with what Perea (2019) has found. An adept command of private speech suggests a sound awareness of planning in language learning, and explicating the learners' frequent use of meta-cognitive learning strategies (Hu and Gao, 2018).

Social agents including teachers and peers were found to have a mediational effect on the CSL learners' deployment of learning strategies, particularly social-affective strategies. This result lends support to a number of previous studies (e.g., Li, 2014; Gong et al., 2021b). There are two possible reasons explicating the mediating role of teachers in shaping the learners' use of affective strategies. For one reason, teacher immediacy seems to shorten the distance between teachers and the CSL learners, as the Interviewees like Doha and Hadi expressed that they enjoyed the opportunities to interact with their Chinese language teachers in case they encountered problems in study and suffered from hardships in life. Thus, a rapport is created between the teacher and the CSL learners, which contributes to their use of affective strategies (Santana-Quintana, 2018). For another, the mediating role might be associated with the teaching beliefs of these native Chinese-speaking teachers. All the CSL teachers hold a qualification in teaching Chinese to speakers of other languages, which means that they are well-prepared in pedagogical theories and beliefs (Ma et al., 2017). One of such beliefs beholds the value of teaching students how to fish (Wei, 2012). This belief praises the training and cultivation of the students' indirect strategic awareness such as meta-cognitive and social-affective strategies.

Another critical social agent relates to peers who were found to exert a mediating effect on the use of meta-cognitive and social strategies among the CSL learners. This result is assonated with previous studies (e.g., Hajar, 2017). The interviewees' frequent use of mate-cognitive and social strategies illustrates their awareness of using indirect strategy for learning Chinese. This strategic awareness, apart from being nurtured from the pedagogical practice of the university, might also result from the availability of native Chinese-speaking peers in the learners' CSL class. The surveyed university provided two teaching assistants and two interim TCSOL postgraduates for each class. These teaching assistants form social resources for the CSL learners. These rich social resources thus offered opportunities for the CSL learners to socialize with the native Chinese-speaking peers, nurturing their strategic awareness and competence for Chinese learning.

A third mediating factor pertains to cultural artifacts associated with the medium language of communication, textbooks and online learning resources, and assessment of high stake examinations. Medium language of communication is found to be used as a compensatory technique to accommodate the limited Chinese competence among the CSL learners like Doha and Ahao. This phenomenon reflects the emerging topic of translanguaging and English as lingua franca against the multilingual background (Li, 2018). The CSL learners originated from diversified linguistic backgrounds. Once faced with difficulty in speaking Chinese, they would translanguage back to their mother tongue or English, an international language.

WhatsApp and other online resources were found of mediational effect upon the use of cognitive and compensation strategies in the cases of the CSL learners like Xiaoye and Benliyi. The online resources provided the CSL learners with a rich variety of Apps and databases for Chinese learning. For example, the CSL learners with access to online resources could learn how to enrich their ways of learning Chinese and compensate for the communication breakdowns caused by their poor language proficiency. Additionally, teaching materials like textbooks were found with a mediating effect on the learners' use of cognitive strategies. This verified the mediating effect of textbooks and notes in second and foreign language learning (Perea, 2019). The textbooks and other teaching materials were compiled with multi-modal features like colorful graphs and videos to accommodate the needs of CSL learners. These multi-modal features could arouse the learners' interest and enrich their repertoire of cognitive strategies in Chinese learning.

Assessment methods were another artifact mediator identified in the participant's deployment of learning strategies. This result verifies some previous findings (e.g., Saks and Leijen, 2018). The surveyed university adopts formative assessment in the evaluation of the CSL learners, including classroom presentations, mid-term examinations, projects, and final examinations. The CSL learners have to sit for the HSK tests, should they expect to obtain degree or scholarship in Chinese universities. These formative elements consequently lead the participants to pay much attention to the learning process and seek effective ways to improve their performance through memorizing more characters, adopting more diversified methods, reflecting, and scheduling their study (Li, 2014). Therefore, their strategic competence is strengthened, which is mainly displayed in their use of memory, cognitive and meta-cognitive strategies among the CSL learners such as Molly, Xiaoyi, and Anan.

Learning environment was found to be the fourth mediator in the learners' use of learning strategies, which accorded with a number of previous findings (e.g., Cruz and Pariña, 2018). The CSL learners' deployment of social (Ahao and Xiaoyi) and meta-cognitive (Yiwan) strategies was mediated by the social environment, as they expressed that they learned Chinese by interacting with local people. It seemed that the participants have realized the value of native target language contexts for learning Chinese. This strategic belief echoes the perception of the primacy of learning a foreign language in the native target language context, which prevails among second/foreign language learners (Li, 2021). This is because in the target language environment like China, there are abundant social resources such as native Chinese speakers for Chinese learning. These rich social resources thus provide adequate opportunities for the CSL learners to interact with the locals using Chinese (Gong et al., 2021a).

Situational learning environment like classroom environment and extracurricular associations is another mediator of learning environment, which has been found to mediate the use of social-affective strategies among learners like Doha, and Xiaoye. This finding corroborates with some previous studies (e.g., Li, 2014; Huang, 2018). This might attribute to the opportunities created by the surveyed university for the CSL learners to interact with local Chinese students. For each class, the university offered Chinese students as teaching assistants to help the CSL learners. Besides, there were campus clubs and societies such as Chinese Calligraphy Association, and Chinese Bridge Series of Chinese Proficiency Competition. All these extra-curricular activities create numerous channels for the learners to immense themselves into the Chinese learning environment. Therefore, the CSL learners could easily reach out to the local Chinese students for interaction. This involvement would thus affect the learners' emotional state and increase their use of social-affective strategies (Li, 2014).

The present findings also indicate that the adoption of Chinese language learning strategies is the result of the interplay between learner self and the situational, institutional, and contextual factors. This finding aligns with the studies by Li (2014) and Gao (2010), which demonstrated that L2 learners displayed a wide repertoire of learning strategies when relocated into the target language settings. The strategic use of the learners was simultaneously influenced by personal and contextual factors. To be concrete, the learners internalized the value of learning a second/foreign language in the target language environment through immersion into the Chinese-speaking context, interaction with the local Chinese people, and their learning experiences in class and extra-curricular activities. The internalization of these situational and contextual realities seems to shape their strategic beliefs, which indicates their exercise of agency in CSL learning. Their agentic power is also reflected in their use of private speech and their ideal L2 self (such as the reflection in Ahao's motivation), which tends to influence their approbation of learning strategies.

## Conclusion

The present study explored the strategy use and its mediating factors among a group of CSL learners in mainland China from the mediation theory perspective. It is found that the surveyed participants displayed a wide orchestration of strategies in their Chinese learning. The present study verifies previous findings that social and meta-cognitive strategies are most frequently deployed by the learners in both the CFL and CSL contexts. It also gains new insights into strategy use in that memory strategies are the most frequently used, and that affective strategies are moderately used among the CSL learners, which contradicts previous finding. Their approbation of learning strategies is mediated by an array of personal, situational, institutional, and contextual factors. Specifically, the learners' deployment of learning strategies was influenced by a series of factors, including learner self such as private speech and ideal L2 self, social agents like teachers and peers, artifacts including medium language of communication, assessment methods, and online resources, societal environment and extra-curricular activities. The present study contributes to existing literature in the following aspects. It firstly enriches the literature on strategy use of CSL learners. Besides, this study validates the feasibility of integrating the mediation theory into the exploration of L2 learning strategy, which consequently provides a new theoretical approach for L2 strategy research. Furthermore, it illuminates the contextually situated nature of strategy use, which sheds light on the interplay between learner agency and socio-cultural conditions that mutually shapes the learners' strategy use in their Chinese learning.

The present findings are implicative for teaching Chinese to speakers of other languages in China and other similar contexts. This study found that teachers are the most critical agents in mediating the learners' strategic use. This finding informs the development of teachers of non-Chinese learners against the background of the rapidly increasing number of Chinese language learners and educators (Ma et al., 2017). Teachers need to sensitize themselves of the strategy profiles of their students (Zhang and Wan, 2019). In light of the mediating effect of artifacts, CSL/CFL teachers are suggested to integrate the updated Apps and other online resources for Chinese learning into their class. This study also indicates the necessity and importance of a collective effort from teachers, policy makers and administrators to create a Chinese-speaking context for the learners. In view of the contributing effect of agentic power, it is suggestive that strategy-based instruction be provided to train the learners so as to enlarge their strategic repertoire. In addition, considering the diversity in cultural backgrounds of the Chinese learners and the profound mediating effect of teachers upon the learners' practice of Chinese learning (Gong et al., 2021b), it is essential for teachers to strengthen their intercultural competence.

This study has some shortcomings. First of all, this study only followed a cross-sectional paradigm to investigate the features and influencing factors of strategy use among the participants. Besides, this study did not take into account other learner variables such as language proficiency and length of time spent learning Chinese. Therefore, it is cautioned when generalizing the findings of this study into other contexts. Future studies are called for to adopt a longitudinal paradigm with larger sample population from various institutions. It is also recommended to further explore the interrelationship between strategy use and other factors of CSL learners with diverse language proficiency levels and learning experiences.

## Data Availability Statement

The original contributions presented in the study are included in the article/supplementary material, further inquiries can be directed to the corresponding author/s.

## Ethics Statement

The studies involving human participants were reviewed and approved by School of Foreign Languages, Hubei University of Technology, China. The participants provided their written informed consent to participate in this study.

## Author Contributions

CL designed the study and finalized the draft. LC collected, analyzed, and made the first draft of the manuscript. CM helped process the data. SZ analyzed and helped revise the manuscript. HH helped revise the manuscript. All authors contributed to the article and approved the submitted version.

## Funding

The authors are grateful for the support of this study from the following funds: The National University Foreign Language Teaching and Research Fund (No. 2018HB0088A), The Social Sciences Research Fund of Hubei Provincial Department of Education (No. 18Y067), The Humanities and Social Sciences Fund of Hubei University of Technology (No. 2018SW0303), and The Teaching and Research Fund of Hubei University of Technology (No. Xiao2018025).

## Conflict of Interest

The authors declare that the research was conducted in the absence of any commercial or financial relationships that could be construed as a potential conflict of interest.

## Publisher's Note

All claims expressed in this article are solely those of the authors and do not necessarily represent those of their affiliated organizations, or those of the publisher, the editors and the reviewers. Any product that may be evaluated in this article, or claim that may be made by its manufacturer, is not guaranteed or endorsed by the publisher.

## Appendix 1

Interview protocol.

1. Do you like Chinese?

2. How do you usually learn Chinese? Could you give any example?

3. What do you usually do when you have a problem in learning Chinese? Could you give any example?

4. Could you describe a Chinese class that impresses you at this university?

5. What do you think of the role of examinations in your Chinese learning, like HSK? Why or why not?

6. How do you think of the role of other people like your teachers, peers, and family members in your Chinese learning? Why or why not?

7. How do you learn Chinese after class? Do you participate in any Chinese activities after class? Why or why not?
